# A Full-Scale Agent-Based Model to Hypothetically Explore the Impact of Lockdown, Social Distancing, and Vaccination During the COVID-19 Pandemic in Lombardy, Italy: Model Development

**DOI:** 10.2196/24630

**Published:** 2021-09-10

**Authors:** Giuseppe Giacopelli

**Affiliations:** 1 Department of Mathematics and Informatics University of Palermo Palermo Italy

**Keywords:** epidemiology, computational, model, COVID-19, modeling, outbreak, virus, infectious disease, simulation, impact, vaccine, agent-based model

## Abstract

**Background:**

The COVID-19 outbreak, an event of global concern, has provided scientists the opportunity to use mathematical modeling to run simulations and test theories about the pandemic.

**Objective:**

The aim of this study was to propose a full-scale individual-based model of the COVID-19 outbreak in Lombardy, Italy, to test various scenarios pertaining to the pandemic and achieve novel performance metrics.

**Methods:**

The model was designed to simulate all 10 million inhabitants of Lombardy person by person via a simple agent-based approach using a commercial computer. In order to obtain performance data, a collision detection model was developed to enable cluster nodes in small cells that can be processed fully in parallel. Within this collision detection model, an epidemic model based mostly on experimental findings about COVID-19 was developed.

**Results:**

The model was used to explain the behavior of the COVID-19 outbreak in Lombardy. Different parameters were used to simulate various scenarios relating to social distancing and lockdown. According to the model, these simple actions were enough to control the virus. The model also explained the decline in cases in the spring and simulated a hypothetical vaccination scenario, confirming, for example, the herd immunity threshold computed in previous works.

**Conclusions:**

The model made it possible to test the impact of people’s daily actions (eg, maintaining social distance) on the epidemic and to investigate interactions among agents within a social network. It also provided insight on the impact of a hypothetical vaccine.

## Introduction

The first case of COVID-19 was detected in China [1], but one of the most serious outbreaks occurred in Italy at the end of January 2020 [2]. This epidemic witnessed a change in risk management: the use of mathematical modeling [3]. As mathematical modeling is complex [4], there are many approaches to solving these problems. One such approach is agent-based modeling [5], which in epidemiology has been used widely in the past. However, due to its computational limitations, approaches based on differential equations like SIR (susceptible-infected-recovered) models have often been preferred [6]. In particular, SIR models are typically mediated by ordinary differential equations (ODEs) [7] and have been used to model general populations worldwide [8,9], as well as the entire Italian population in particular [10]. However, ODE models often require many free parameters to be computed, and they cannot usually be derived directly from experimental data because these parameters are abstract quantities. Hence, the most common approach to ODE models in epidemiology is to fit all the free abstract parameters to experimental time series that will be explained by the model. However, it is difficult to test and quantify alternative scenarios with this approach since the parameters are very abstract.

To solve these problems, the latest advances in computer science and engineering, as well as the COVID-19 outbreak itself, have led to the use of agent-based models for simulating small community epidemic behaviors since in agent-scale simulations. The parameters, all of which involve the individual, are usually experimentally constrained and determined. Previous work by Gharakhanlou and Hooshangi [11] explored the COVID-19 outbreak using an agent-based model of approximately 750,000 inhabitants in the city of Urmia, Iran, with the movement of agents approximated by their location. Similarly, Son et al [12] used a transmission model with a subsampled population of 9000 people living in Daegu, South Korea. There are small-scale models as well, as shown by Cuevas [13]. Also worthy of mention is the model developed by the University of Palermo [14], which was based on the work of Muggeo [15].

The aim of this study was to present a qualitative, full-scale agent-based model with the ability to reproduce the COVID-19 dynamics of Lombardy, Italy, modeling its outbreak and decline in cases, including as much real and open-access data as possible. Lombardy’s population of 10.06 million makes this model very large scale compared to previous works. Secondarily, the study aimed to investigate several alternative scenarios in order to assess their impact at the time. Finally, a social interaction model, used in epidemiological simulations, was employed, per graph theory [16], to study the agents’ interactions as a social network [17]. The results were used to draw several conclusions about the impact of people’s habits during the COVID-19 outbreak.

## Methods

### The Model Structure

**Figure 1:**
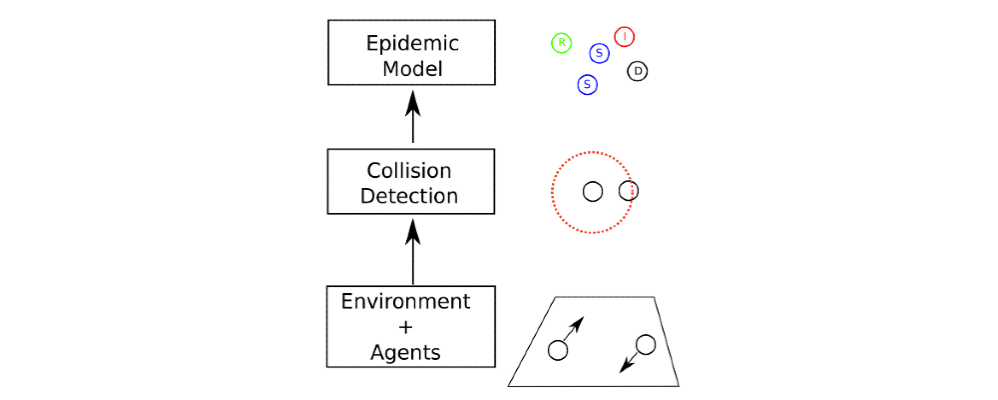
The 3-layer structure of the model. The first layer, environment and agents, represents the motion of the inhabitants. The second layer represents social interaction between people in terms of collision detection. The third layer represents the virus dynamic in terms of epidemic behavior. S: susceptible, I: infected, R: recovered, D: deceased.

The key objective was to create a 3-layer model (Figure 1). The first layer was an agent-based particle model for Lombardy. Every agent is an inhabitant of the region, making this model a full-scale model of Lombardy. Therefore, we have 10.06 million agents who move according to the random walk theory [18]. The random walk behavior must be intended as an approximation of the actual motion of people during the day; this approximation was introduced to reduce the amount of information required to run the model and is widely used in many fields of science (eg, ideal gas theory) [18]. The large number of agents simulated is part of the novelty of this study because (to the best of the author’s knowledge) it is the first to attempt to simulate such a large population individual by individual for this purpose.

A collision detection algorithm was built within the agent-based model to detect whether 2 particles have a distance less than a fixed value. However, the large scale reached by the model required an ad hoc algorithm for this purpose; this challenging problem was solved via a square cells algorithm that permits the code to run in parallel (thereby decreasing the computational complexity of the task).

An epidemic model was built within the collision detection model, that is, a susceptible-infected-recovered-deceased (SIRD) model [19]. The model was filled with many experimental findings on COVID-19, and some fitting parameters were tuned on experimental findings. The whole analysis used as much open-access research as possible; moreover, the entire project was fully open source and is available on GitHub [20].

The model comprised three different layers:

1. The environment and agents model allows for the use of real data in agent movement, creating the first difference between the proposed model and ODE-based models [6] since this kind of model often only uses a few equations to describe large populations, which makes it difficult to take into account ensemble observations on single agents. On the other hand, the proposed model is suitable for a large number of agents, differentiating from previous contributions in this field [11,12].
2. The collision detection model, via 2-km–sided square cells, allows the code to run in parallel, making it possible to compute the epidemic spread of a population of 10 million people agent by agent.
3. The agent-based epidemic model, based on the Markovian process [19], makes it possible to use the experimental probability of infection measured directly from experimental data. This allows the model to be suitable for evaluating alternative scenarios in contrast to ODE-based models [6], which usually must be tuned to fit the time series observed.

### The Agents Model

The agents model simulates the behavior of each inhabitant of Lombardy using the approximation of random walks [18]. The displacement of the particles follows the density of inhabitants in Lombardy (ie, publicly available data). Even if more accurate data on people displacement and movement could be used, privacy concerns may not permit the open-source and open-access distribution of this data. Per the random walk approach, every particle moves with a random vector at every step. The model runs at 6 frames per day, which is a good frame rate considering that the scale time for epidemic phenomena is usually months; however, this can be improved as discussed in the Conclusions section. The random walk approach can appear unrealistic, but this hypothesis has been shown to be appropriate to model very large-scale systems (eg, gas thermodynamics [21]). In addition to random walks, a weak velocity field with a dependence of 1/*r*^2^ was added*,* where *r* is the distance between 2 particles, as in a gravitational field, in order to aggregate the particles. The drift speed of the particles is constant and selected with a Weibull distribution [22] with a scale parameter of 6 and a shape parameter of 1.5. The particles’ speeds were adjusted through a multiplicative constant in order to make the average path length of a particle in a day about 43 km, as suggested in a report by UnipolSai Assicurazioni [23].

### The Environment Model

The setting of the simulation is Lombardy, making the environment model a closed 2D box with a boundary shape following Lombardy’s borders. In order to keep the particles inside the region, a bouncing condition was introduced at the border, so that a particle that tries to cross the border bounces backward. This condition is very popular in gas thermodynamics [18]. The initial conditions of the particles in terms of position are distributed following the actual density of the population of Lombardy, extracted from UnipolSai Assicurazioni [23] via image analysis [24]; this is then intended as an approximation of the real data.

### Collision Detection

Starting with the assumption that the algorithm has been designed to run on a commercial computer in parallel (the one used in the study has AMD 3900X 12-cores and 64 GB of RAM) and within reasonable time (about 20 minutes of calculation for 14 days of simulation), the collision detection algorithm played a central role in the implementation of the algorithm. In order to find all points with a distance less than a constant in a set of *N* points, a complexity order of *N*^2^ is generally needed. In our model *N*≈10^7^, the complexity order was 10^14^, which is a large number.

Next, Lombardy was subdivided into a grid, 20 km in dimension. Collision detection was applied to every cell of the grid, and every cell was assigned to a separate parallel job to run the computation in parallel through the cells. This multiscale processing allowed for the speeding up of the code, reducing the RAM used simultaneously in computation, which made possible a simulation with 10 million particles at the same time. This approach neglects all the connections across the borders of the cells, but this is beyond the aims of this study.

The creation of this algorithm was a challenging aspect of this study. The idea was to use matrix optimization in order to speed up the computation. The territory was subdivided into 20-km–long cells, and the cells in every frame were completely independent, with the supposition that, on average, every cell contains *m* people. In order to compute the distance between all nodes in the network, we had to compute the order of *N*^2^ pairwise distances.

With this scheme, we had to only compute the order of *m*^2^ distances for each block multiplied by the number of blocks (which is about *N/m*) that is an order of *Nm*. Considering *m* small in comparison with *N*, it can be said that the scheme has a complexity near the order of *N* (for large *N* and small *m*). However, determining in which cell a person is located was also challenging because of the large size of the population. For these reasons, a simple grid scheme was used to locate nodes inside the cells. We used the following idea—supposing a segment from 0 km to *L_C_=*2 km with *N_c_=*4 cells:

1. From 0 km to 0.5 km
2. From 0.5 km to 1 km
3. From 1 km to 1.5 km
4. From 1.5 km to 2 km

If, for example, the point *p*=0.6 km needed to be located, the formula used to calculate this would be *id_p_*=*ceil*(*N_c_p*/*L_c_*). The result is 2, indicating the second cell. Applying this formula for the x-axis and y-axis allows the algorithm to locate people in the cells. Although this algorithm may appear to be simple, it requires few calculations to be computed, which can make a substantial difference when a large number of agents is concerned.

### The Epidemic Model

The epidemic model is an SIRD model [3]. Most of the models available up to now are called population models [25]. A population model is a model where every node is modeled by a set of differential equations; it models a subpopulation of a region. The number of people modeled by a single node can range from hundreds to millions. In our model, every node is a single person. The model is not an ODE model, but a stochastic agent-based model. Every node has four states:

1. Susceptible: a node that has not already contracted the disease. It can be become infected with a probability *p_I_* for each contact with an infected node;
2. Infected: a node that is infected, which can then infect susceptible nodes. After *E* days, this node will change its state to recovered or to deceased, with a probability *p_D_* to die and 1–*p_D_* to recover;
3. Recovered: a node that has recovered from the disease and cannot contract it or infect susceptible nodes anymore;
4. Deceased: a node that has died and hence cannot infect other nodes.

### Validation

**Figure 2:**
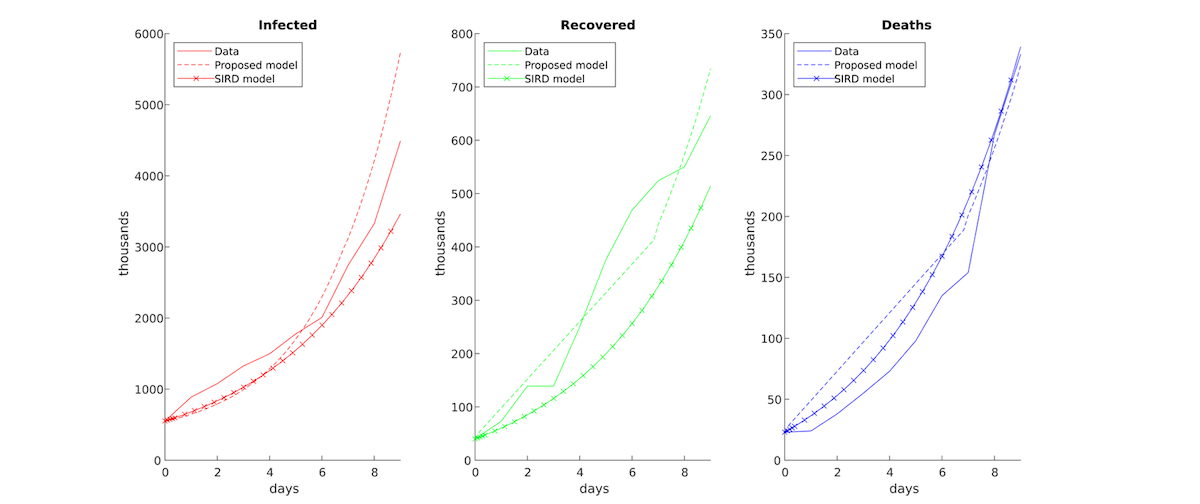
Comparison between data on the outbreak, the proposed model, and a classical susceptible-infected-recovered-deceased (SIRD) model [26].

The proposed model was compared with a classical SIRD model [26] fitted with a parameter exploration scheme on outbreak data (Figure 2). As seen in Figure 2, the results are comparable in terms of the rooted mean square error of the data: the SIRD model had an error of 150 for the infected, 71 for the recovered, and 18 for the deceased; and the proposed model exhibited an error of 535 for the infected, 58 for the recovered, and 34 for the deceased. This indicates that our model has comparable performance with the SIRD model (outperforming in the recovered), but it is not ODE mediated and is thus suitable for testing alternative scenarios. Moreover, in this paper, since most of the parameters are realistic, the model can be run for a general epidemic upon collecting the few parameters required (which in this case were all open access) and fitting the two parameters left. However, the model can be made more precise by adding additional realistic data, which most of the time are not fully open access; this, however, is out of the scope of this study.

## Results

All simulations are available in .avi format on GitHub [20], as well the MATLAB code, for reproducibility.

### The Lombardy Outbreak

The first scenario was the Lombardy outbreak of March 2020 [2]. Our simulation began on February 29, 2020, and terminated on March 14, 2020. The main realistic parameters were *p_I_*=1/40,500 (extracted from Bhatia and Klausner [27]) and *p_D_*=0.3 (estimated from Worldometer [28], which has also been cited by Dhillon et al [29]).

The fitted parameters have a collision radius of 1 km. This can appear very large compared to the 1-m distance suggested by the World Health Organization [30]; however, when taken into account that there are 6 frames per day, then 1 km is the radius of the interaction of a person who remains in the same place for 4 hours and the duration of the disease (in days) *E*=7 (the Centers for Disease Control and Prevention suggests *E*<10 [31]).

**Figure 3:**
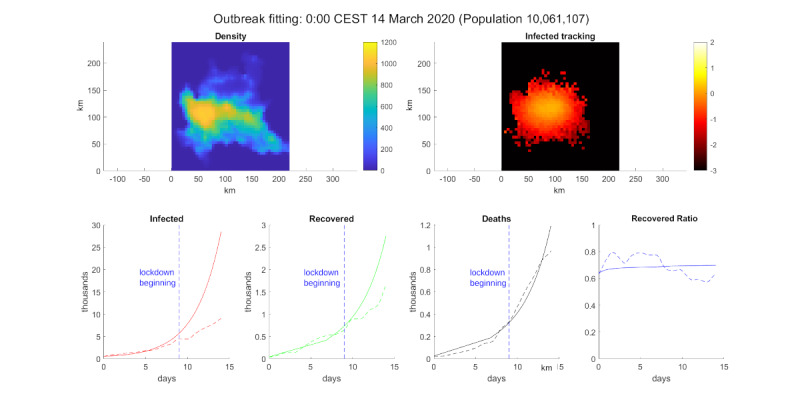
COVID-19 outbreak simulation. Top-left: population density. Top-right: log_10_ of the infected percentage per cell. Bottom, from left to right: infected number, recovered number, deceased number, and recovered ratio (recovered/deaths). The solid line is the model simulation, the dotted line is extracted data from the Ministry of Health/Civil Protection Department [33] for Lombardy, and the vertical dotted blue line marks the date March 9, 2020 [32].

The results of the simulations can be seen in Figure 3. The model was able to explain the experimental data until approximately March 9, 2020. On this day, the Decree of the President of the Council of Ministers (DPCM) implemented measures to contain the COVID-19 outbreak [32], which included the beginning of the lockdown in Italy. This discrepancy between the data and the model was the result of the collective effort of the Italian populace to protect itself against SARS-CoV-2. Therefore, the simulation serves to provide a warning about what could have happened.

### Impact of People’s Habits

**Figure 4:**
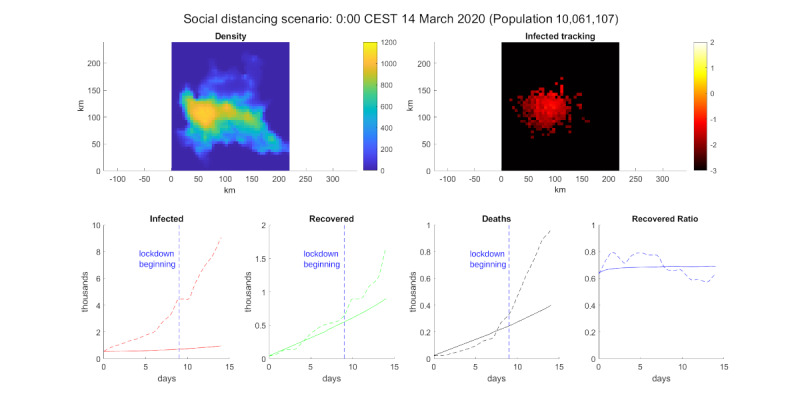
Social distancing simulation. Top-left: population density. Top-right: log_10_ of the infected percentage per cell. Bottom, from left to right: infected number, recovered number, deceased number, and recovered ratio (recovered/deaths). The solid line is the model simulation, the dotted line is extracted data from the Ministry of Health/Civil Protection Department [33] for Lombardy, and the vertical dotted blue line marks the date March 9, 2020 [32].

The second scenario was inspired by Chu et al [34], who showed that maintaining a 2-m distance between people halved the risk of contracting COVID-19. Thus, we aimed to simulate this kind of social distancing by halving *p_I_* in the model. The results (Figure 4) showed that COVID-19 (in this scenario) was not contagious enough to spread as in the experimental data. This simulation demonstrated the striking role of a simple action like social distancing in fighting COVID-19 and highlighted the difference between a virus under control and a disease of epidemic proportions. This simple fact has already been observed in experimental findings in Germany [35], where a synthetic method was used to estimate the spread of the contagion without the use of masks.

**Figure 5:**
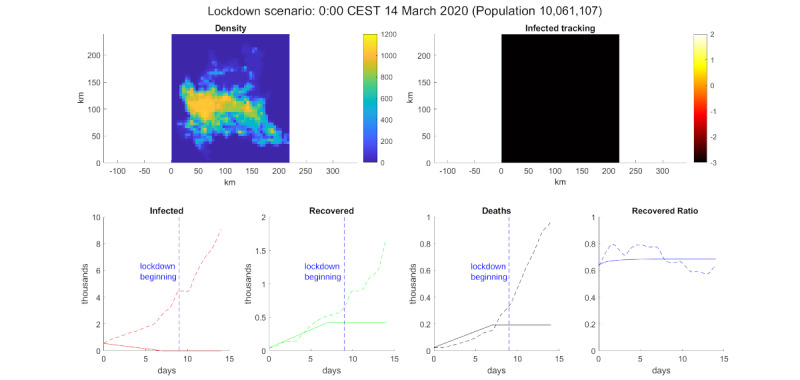
Lockdown simulation. Top-left: population density. Top-right: log_10_ of the infected percentage per cell. Bottom, from left to right: infected number, recovered number, deceased number, and recovered ratio (recovered/deaths). The solid line is the model simulation, the dotted line is extracted data from the Ministry of Health/Civil Protection Department [33] for Lombardy, and the vertical dotted blue line marks the date March 9, 2020 [32].

We also performed a lockdown simulation, reducing the daily average kilometers traveled by a node from 43 km to 5 km and reducing the interaction distance from 1 km to 100 m. The results of this simulation can be seen in Figure 5. According to the model, these simple actions were enough to control the virus.

### Network Topology

**Figure 6:**
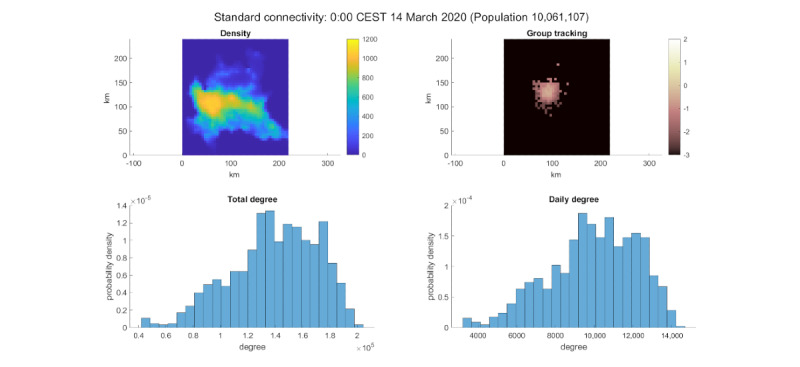
COVID-19 outbreak simulation connectivity. Top-left: population density. Top-right: log_10_ of the group percentage per cell. Bottom-left: degree distribution of the test group. Bottom-right: daily degree distribution of the test group.

The impact of topology in an epidemic model is a popular topic [30,36] in the debate on social networks. Thus, we performed a test: 1000 particles were chosen and then tracked across all simulations to find the total number of connections (ie, contact between particles) made within the whole population. In graph theory, the number of connections of a node is called a degree [16]. This test allowed us to determine the degree distribution and the daily degree distribution (average degree per day) of this small group of people across time. However, only the final result is presented (the full simulation is available on GitHub [20]). The first scenario was the COVID-19 outbreak scenario (Figure 6).

**Figure 7:**
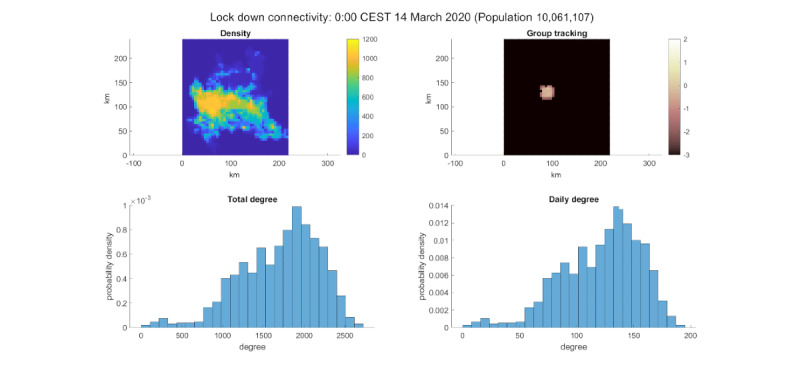
Lockdown simulation connectivity. Top-left: population density. Top-right: log_10_ of the group percentage per cell. Bottom-left: degree distribution of the test group. Bottom-right: daily degree distribution of the test group.

It can be seen that the distribution has an evident left tail (in contrast with the right tail of the Barabási-Albert models [17]). This was probably due to the simulation time of 14 days (in contrast with human social networks, which usually take years to be built). The lockdown scenario was also interesting. In this scenario, we observed a decline in connectivity from thousands of average connections per day to hundreds (Figure 7). This shows the importance of lockdowns in COVID-19 containment.

### A Decline-in-Cases Scenario

**Figure 8:**
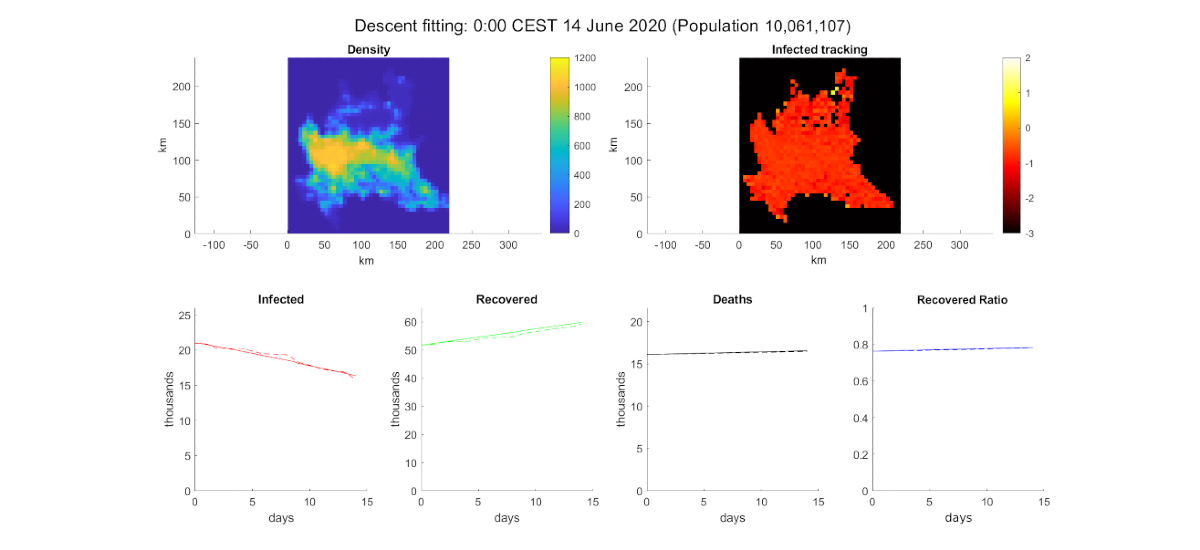
Simulation of a decline in cases. Top-left: population density. Top-right: log_10_ of the infected percentage per cell. Bottom, from left to right: infected number, recovered number, deceased number, and recovered ratio (recovered/deaths). The solid line is the model simulation and the dotted line is extracted data from the Decree of the President of the Council of Ministers [32] for Lombardy.

This scenario took into account the period between May 31, 2020, and June 14, 2020. During this period, Italy concluded its lockdown, and the number of active cases was decreasing. For this simulation, the kilometers per day was set arbitrarily to 15 km because of the lack of additional information on mobility during this period. The probability of contracting the contagion was halved to account for social distancing. The radius of interaction and the duration of the disease were tuned to reproduce the experimental data. The value for the radius of interaction was 300 m and disease duration was 5 weeks (*E*=35). This value (which is higher in comparison to that of the outbreak) could be influenced by a clinical protocol more accurately and by the queue created by the large number of infected people, which could slow down the tests required to declare recovery. The qualitative fitting can be seen in Figure 8.

### The Vaccine Scenario

**Figure 9:**
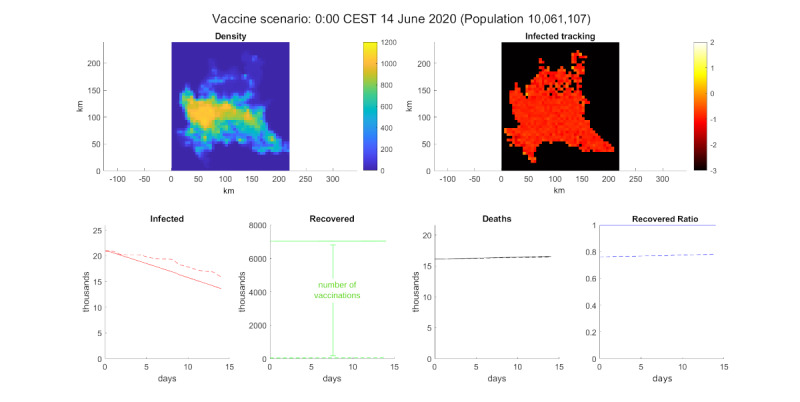
Simulation of vaccination. Top-left: population density. Top-right: log_10_ of the infected percentage per cell. Bottom, from left to right: infected number, recovered number, deceased number, and recovered ratio.

Using the previous scenario of a decline in cases, we tested the impact of vaccinating 70% of the population, similar to the 62% suggested by Park and Kim [37]. The agent-based models are suitable for testing strategies like vaccination at the individual level. The result of the simulation was a strong decrease in infections, which was unexpected in a simulation of 14 days. The results are shown in Figure 9.

## Discussion

### Principal Findings

This model demonstrated the importance of people’s actions in an epidemic setting. Indeed, the behavior of the virus was indicative of our own habits [17]. The agent-based model proposed here has shown great flexibility in simulating alternative scenarios; in contrast, although ODE models [6] are faster than the proposed model, they are not suitable for this task.

### Limitations

The model proposed is more computationally expensive than ODE models, which require the calculation of few differential equations to simulate large populations. In general, such algorithms are also faster than agent-based models. The proposed model, however, allows for the interpretation of complex parameters.

### Comparison With Prior Work

This study has explained the behavior of the COVID-19 outbreak in Lombardy and has validated the herd immunity threshold obtained with different techniques [37], even if the 62% proposed by Park and Kim [37] is less than the 70% proposed in this study. This contribution also provides a new methodology in social network analysis, where the graph theoretical approach is substituted by agents. It also paves the way to more realistic epidemic models, where hypothetical scenarios can be tested directly on the agents, without any ODE mediation.

### Conclusions

This work provides a novel, efficient, and low-demanding (in terms of computational resources) population model. Many features remain to be introduced in the model, like an age-dependent virus model, the ability to introduce an age parameter in the model or a more precise spatial simulation based on big data, and the ability to simulate the habits of the population. In conclusion, future work could be done to increase the number of frames per day, thereby improving the performance of the agents.

## Abbreviations

DPCM: Decree of the President of the Council of Ministers
ODE: ordinary differential equation
SIR: susceptible-infected-recovered
SIRD: susceptible-infected-recovered-deceased
